# Isolated gastrointestinal Langerhans cell histiocytosis in a 16‐month‐old child: A case report

**DOI:** 10.1002/jpr3.70096

**Published:** 2025-10-14

**Authors:** Al‐Qasim AL‐Bahlani, Laraib Touseeq, Mohammed Al‐Masqari, Emad Saad

**Affiliations:** ^1^ Department of Child Health, Pediatric Gastroenterology and Hepatology Section Royal Hospital Muscat Sultanate of Oman; ^2^ Histopathology Department Royal Hospital Muscat Sultanate of Oman; ^3^ National Oncology Centre Royal Hospital Muscat Sultanate of Oman

**Keywords:** BRAF mutation, chronic diarrhea, histological diagnosis, rare pediatric gastrointestinal disease

## Abstract

Langerhans cell histiocytosis (LCH) is a rare disorder characterized by clonal proliferation of Langerhans cells, most often involving the skin or bone. Isolated gastrointestinal (GI) involvement is extremely uncommon in young children. We report a 16‐month‐old girl with a 1‐month history of chronic vomiting, bloody diarrhea, and failure to thrive who was found to have GI‐LCH without systemic involvement. She was started on standard LCH chemotherapy with a favorable clinical response. This case highlights the importance of considering LCH in infants/toddlers with unexplained GI symptoms and the utility of targeted molecular testing (e.g., BRAF mutation analysis) in guiding therapy.

## 1 INTRODUCTION

Langerhans cell histiocytosis (LCH) is a rare pediatric histiocytic disorder with diverse clinical presentations. It can range from an isolated single‐organ disease to disseminated multisystem involvement. Activating mutations in the mitogen‐activated protein kinase (MAPK) pathway, most frequently the BRAFV600E mutation, are identified in the majority of LCH cases. BRAFV600E occurs in approximately 50% of pediatric patients and is associated with more aggressive disease (including multisystem and “risk organ” involvement) and higher risk of treatment failure.^1^, ^2^ We present an infant/toddler with isolated gastrointestinal LCH (GI‐LCH), emphasizing the diagnostic challenges and management approach, including the potential role of targeted therapy.

## 2 CASE REPORT

A 16‐month‐old female presented with a 1‐month history of frequent vomiting and diarrhea with blood streaks in the stool, accompanied by intermittent fever and poor weight gain. She required multiple hospital admissions for dehydration; her weight remained 7.4 kg (below the third percentile) over 4 months. There were no cutaneous lesions, oral ulcers, joint swelling, or perianal lesions, and her family history was unremarkable.

On examination, the child appeared irritable and cachectic. There was no clubbing, skin rash, lymphadenopathy, hepatosplenomegaly, ascites, or jaundice. Initial laboratory investigations showed hemoglobin 10.6 g/dL with microcytosis (MCV 72 fL), white blood cell count 6.2 × 10^9^/L, and platelet count 839 × 10^9^/L (reactive thrombocytosis). Inflammatory markers were discordant (erythrocyte sedimentation rate 42 mm/h with C‐reactive protein <4 mg/L). She had profound hypoalbuminemia (albumin 20 g/L), suggesting protein‐losing enteropathy, while HIV serology and immunoglobulin levels were normal. Stool cultures and *Clostridium difficile* toxin assay were negative. Fecal calprotectin was markedly elevated at 635 mg/kg (normal < 50), indicating significant intestinal inflammation.

**Figure 1:**
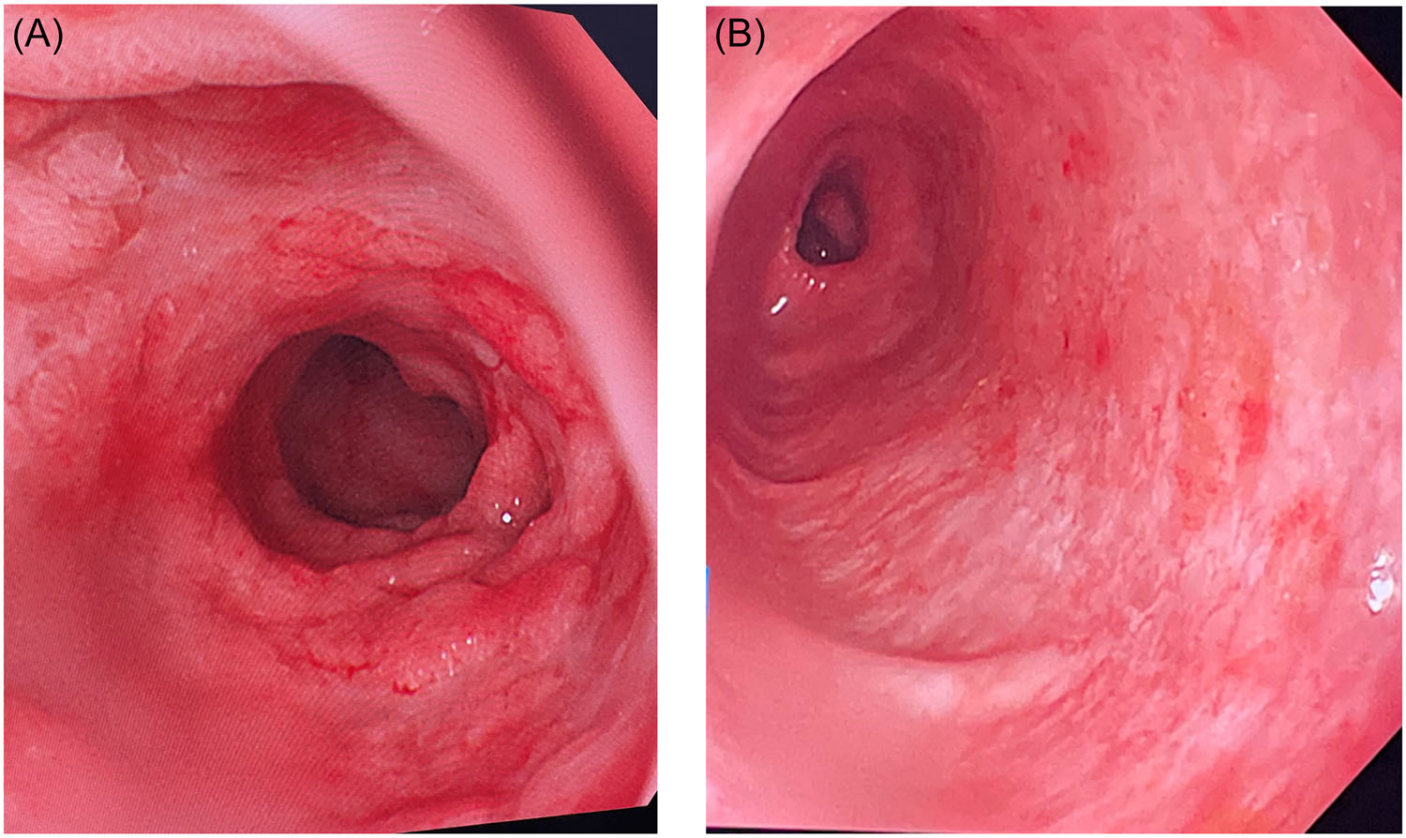
Endoscopic images of the duodenum and colon showing diffuse mucosal nodularity and ulcerative lesions. Endoscopic findings at initial presentation. (A) Duodenum showing mucosal nodularity, edema, and ulceration. (B) Colon showing loss of vascularity, erythema, and erosions.

Abdominal ultrasound showed mild thickening of the right colonic wall with reactive mesenteric lymphadenopathy. Upper endoscopy revealed diffuse duodenal mucosal erythema, edema, nodularity, and scattered small ulcerations. Colonoscopy showed loss of the normal vascular pattern and generalized nodularity with numerous slightly elevated erythematous lesions throughout the colon and rectum; the terminal ileum appeared normal (Figure 1).

**Figure 2:**
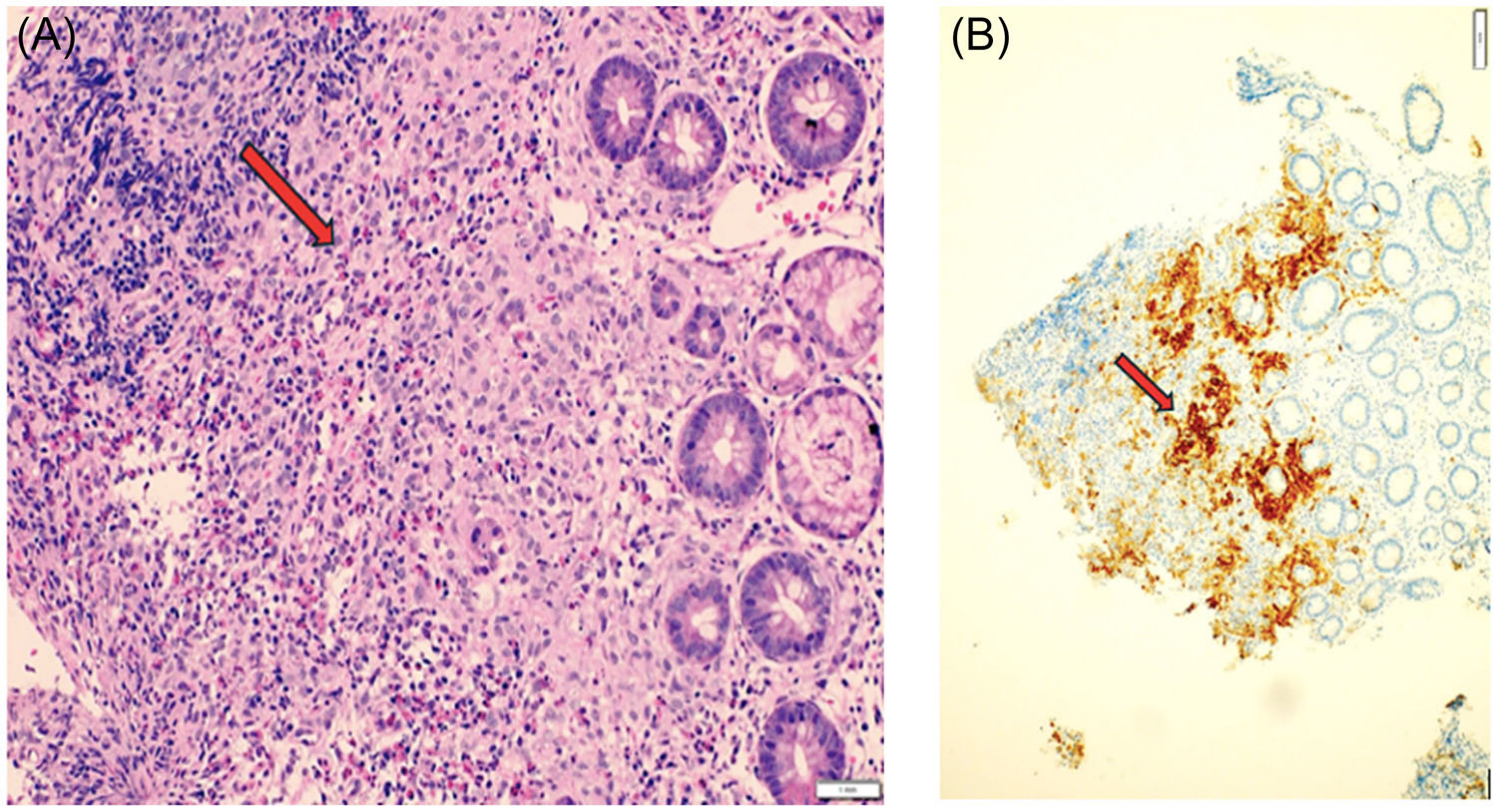
Histopathology of colonic biopsy showing infiltration by Langerhans cells with characteristic nuclear features. (A) Duodenal biopsy. Intermediate power view (20×) showing expansion of lamina propria by Langerhans cells admixed with numerous eosinophils. (B) duodenal biopsy. Low power (4x) demonstrates positive CD1a immunohistochemistry in Langerhans cells.

Multiple endoscopic biopsies (from stomach, duodenum, colon, and rectum) demonstrated sheets of abnormal Langerhans cells infiltrating the mucosa. On immunohistochemistry, the lesional cells were strongly positive for CD1a and S‐100 (with focal CD68 positivity), consistent with LCH. Molecular testing identified the BRAFV600E mutation in the colonic lesion, confirming the diagnosis of isolated GI‐LCH. Further evaluation for systemic involvement, including whole‐body MRI, skeletal survey, and bone marrow aspirate, revealed no evidence of disease elsewhere (Figure 2).

The patient was treated as per the LCH‐IV protocol, Stratum I, Group 1. She received initial Course 1 (vinblastine and prednisone for 6 weeks) and initial Course 2 for another 6 weeks. Continuation therapy was started at Week 14 as per CT‐Arm D (prolonged, 24‐month continuation therapy, with a regimen consisting of vinblastine, prednisone, and 6‐mercaptopurine). After 3 months of treatment, a follow‐up endoscopy showed resolution of the gross lesions, although biopsies still demonstrated persistent LCH cells. Clinically, the child's condition improved with cessation of vomiting, normalization of stool frequency, and notable catch‐up growth.

She completed 24 months of chemotherapy as per protocol. A follow‐up upper and lower endoscopy was performed 3 months after completion of therapy (at 27 months from diagnosis), which showed normal mucosal appearance endoscopically. Histopathological examination revealed no residual Langerhans cell infiltrates, confirming complete remission of the disease.

## 3 DISCUSSION

Isolated GI involvement by LCH is exceptionally uncommon in children. When the GI tract is affected, it is usually in the context of multisystem LCH, often with cutaneous lesions preceding the GI symptoms.^2^, ^3^ Most reported cases of GI‐LCH occur in very young children, typically infants/toddlers under 2 years of age.^3^, ^4^ Common clinical manifestations include protracted vomiting, diarrhea (which can lead to malabsorption and protein‐losing enteropathy), abdominal pain, and hematochezia.^2^, ^3^ Given its rarity and nonspecific presentation, GI‐LCH poses a diagnostic challenge and is frequently misdiagnosed as more common conditions (such as inflammatory bowel disease or food protein enteropathy) on initial evaluation.^5^ Early endoscopic examination with adequate biopsies is therefore crucial in infants/toddlers with unexplained GI bleeding, chronic diarrhea, or failure to thrive, as it allows for histopathological confirmation of this rare entity.

Published case series have highlighted the historically poor prognosis of GI‐LCH. Mayumi et al. noted that GI involvement in LCH often coincides with high‐risk features—multisystem disease and severe protein‐losing enteropathy—which contribute to an elevated mortality rate.^3^ Consistently, Yadav et al. reported that more than 50% of affected children (particularly those under 2 years old) died within 18 months of diagnosis.^5^ Our patient represents an unusual case of strictly isolated GI‐LCH without skin or other organ involvement. Notably, she demonstrated a favorable response to first‐line therapy, in contrast to earlier reports of rapidly fatal outcomes. This suggests that early diagnosis and prompt initiation of appropriate therapy can improve the prognosis even in primary GI‐LCH. In our patient, durable remission was confirmed both clinically and histologically after completion of standard chemotherapy, underscoring the potential for full recovery even in isolated GI‐LCH with high‐risk mutations when appropriately managed.

The detection of a BRAFV600E mutation in our patient's lesions has important therapeutic implications. Activating mutations in the MAPK pathway (most commonly *BRAF* V600E) are known drivers of LCH biology.^1^, ^6^ The presence of BRAFV600E is associated with more extensive disease and relative resistance to conventional therapy.^1^ Recently, targeted therapies have emerged as an effective option for refractory or relapsed LCH cases. Inhibitors of BRAF (such as vemurafenib and dabrafenib) and MEK (such as trametinib) have shown high efficacy in inducing remission in patients with risk‐organ involvement or treatment‐resistant LCH.^7^, ^8^ However, disease reactivation is frequently observed when these targeted agents are discontinued, indicating that maintenance therapy may be necessary to sustain remission.^8^ In our patient, the identification of a BRAFV600E mutation provides a clear rationale for targeted therapy in the event of progression or relapse. While she is currently responding to conventional chemotherapy, having a defined molecular target allows for a precision medicine approach if the disease does not fully resolve, highlighting the evolving therapeutic landscape for high‐risk LCH.

## 4 CONCLUSION

Isolated GI‐LCH should be considered in infants/toddlers presenting with unexplained chronic diarrhea, vomiting, and failure to thrive. Early recognition, histopathological confirmation, and molecular studies are crucial for timely intervention.

## CONFLICT OF INTEREST STATEMENT

The authors declare no conflicts of interest.

## ETHICS STATEMENT

Written informed consent was obtained from the patient's legal guardian for the publication of this case report, including all clinical details and associated images.
